# Current perspectives and trends in the treatment of brain arteriovenous malformations: a review and bibliometric analysis

**DOI:** 10.3389/fneur.2023.1327915

**Published:** 2024-01-11

**Authors:** Weixia Tang, Yang Chen, Li Ma, Yu Chen, Biao Yang, Ren Li, Ziao Li, Yongqiang Wu, Xiaogang Wang, Xiaolong Guo, Wenju Zhang, Xiaolin Chen, Ming Lv, Yuanli Zhao, Geng Guo

**Affiliations:** ^1^School of Public Health, Shanxi Medical University, Taiyuan, Shanxi, China; ^2^Department of Neurosurgery, First Hospital of Shanxi Medical University, Taiyuan, Shanxi, China; ^3^Shanxi Provincial Clinical Research Center for Interventional Medicine, Taiyuan, Shanxi, China; ^4^Department of Neurological Surgery, University of Pittsburgh Medical Center, University of Pittsburgh School of Medicine, Pittsburgh, PA, United States; ^5^Department of Neurosurgery, Beijing Tiantan Hospital, Capital Medical University, Beijing, China; ^6^China National Clinical Research Center for Neurological Diseases, Beijing, China; ^7^Department of Emergency, First Hospital of Shanxi Medical University, Taiyuan, Shanxi, China; ^8^Department of Neurosurgery, Peking Union Medical College Hospital, Chinese Academy of Medical Sciences and Peking Union Medical College, Beijing, China

**Keywords:** arteriovenous malformations, bibliometrics, embolization, microsurgery, radiosurgery, therapeutic

## Abstract

**Background:**

Currently, there is a lack of intuitive analysis regarding the development trend, main authors, and research hotspots in the field of cerebral arteriovenous malformation treatment, as well as a detailed elaboration of possible research hotspots.

**Methods:**

A bibliometric analysis was conducted on data retrieved from the Web of Science core collection database between 2000 and 2022. The analysis was performed using R, VOSviewer, CiteSpace software, and an online bibliometric platform.

**Results:**

A total of 1,356 articles were collected, and the number of publications has increased over time. The United States and the University of Pittsburgh are the most prolific countries and institutions in the field. The top three cited authors are Kondziolka D, Sheehan JP, and Lunsford LD. The Journal of Neurosurgery and Neurosurgery are two of the most influential journals in the field of brain arteriovenous malformation treatment research, with higher H-index, total citations, and number of publications. Furthermore, the analysis of keywords indicates that “aruba trial,” “randomised trial,” “microsurgery,” “onyx embolization,” and “Spetzler-Martin grade” may become research focal points. Additionally, this paper discusses the current research status, existing issues, and potential future research directions for the treatment of brain arteriovenous malformations.

**Conclusion:**

This bibliometric study comprehensively analyses the publication trend of cerebral arteriovenous malformation treatment in the past 20 years. It covers the trend of international cooperation, publications, and research hotspots. This information provides an important reference for scholars to further study cerebral arteriovenous malformation.

## 1 Introduction

Brain arteriovenous malformation (bAVM) is complexes of dysplastic vessels, characterized by a nidus with arterial feeders and venous drainage but without intervening capillaries, creating a high-flow, low-resistance channel that shunts blood from arteries to the venous circulation (1). They are prevalent vascular disorders of the central nervous system, with an estimated annual incidence of 1 per 100,000 and a yearly rupture rate of 1%. This can lead to clinical manifestations such as hemorrhage, epilepsy, headaches, progressive neurological deficits, and hydrocephalus (2–4). Although bAVM is relatively rare, they are a definitive cause of intracerebral hemorrhage and high mortality, particularly among children and young adults (5). Advances in non-invasive neuroimaging techniques are gradually increasing the detection rate of asymptomatic bAVM. Treatment strategies for bAVM include conservative management, surgical resection, stereotactic radiosurgery (SRS), and endovascular embolization, either individually or in combination (6). As treatment decisions for bAVM require careful consideration of the risks associated with the natural history of the patient and the intervention, and given the variability in the complexity of bAVM, there is currently no standardized protocol or consensus for their management. This is particularly true for certain bAVM, for which any intervention, whether ruptured or unruptured, may be associated with significant risks. In recent decades, research activities in the treatment of bAVM have significantly increased. These activities cover epidemiology, pathobiology, diagnostic imaging, treatment outcome evaluation, and the development of novel therapies. However, researchers are faced with the challenge of efficiently discerning seminal findings and comprehensively understanding the state of the field as vast amounts of data and literature accumulate. Applying bibliometric methods to assess the status and trends in research on bAVM treatment holds significant value in this context.

Bibliometric analysis is a quantitative research method used to analyze the distribution structure, quantitative relationships and dynamic patterns of literature within a specific academic field or topic. It is used to identify the most active and influential authors, journals, countries, departments and institutions in a given field, as well as prominent publication years. It also assesses the most frequently discussed topics and types of research, helping researchers to identify areas of focus and predict future trends (7). Bibliometric analysis has been widely used in fields as diverse as science policy, health care, environmental science and ecology (8–13), and has become an essential tool for interdisciplinary collaboration. In the field of bAVM treatment research, Runlin Yang et al. used bibliometric analysis to study endovascular treatment of bAVM (14), while Miguel Bertelli Ramos et al. used it to study research on central nervous system arteriovenous malformations (15). However, both studies were limited to analyzing only the top 100 most cited papers, resulting in a relatively small number of publications. In this sense, there may be a limitation in providing a comprehensive review of the knowledge structure and overall landscape in the field of bAVM treatment. Therefore, given the limitations of existing research, this study aims to provide a comprehensive assessment of the developmental hotspots, frontiers and trends in the field of bAVM treatment through bibliometric analysis. The main contributions of this study are: (1) A comprehensive bibliometric analysis of the retrieved literature on bAVM treatment from 2000 to 2022, detailing basic statistical characteristics such as annual metrics, publication categories and sources. (2) An evaluation of the geographical distribution of publications, the construction of international collaborative networks and the analysis of highly cited papers in the field of bAVM treatment research. (3) A variety of keyword analysis techniques, including keyword evolution, co-occurrence, burst detection and temporal visualisation of keyword trends. (4) An in-depth discussion of the current state of bAVM treatment research, challenges within the field, and potential directions for future study.

## 2 Manuscript formatting

### 2.1 Data sources and search strategy

**Figure 1:**
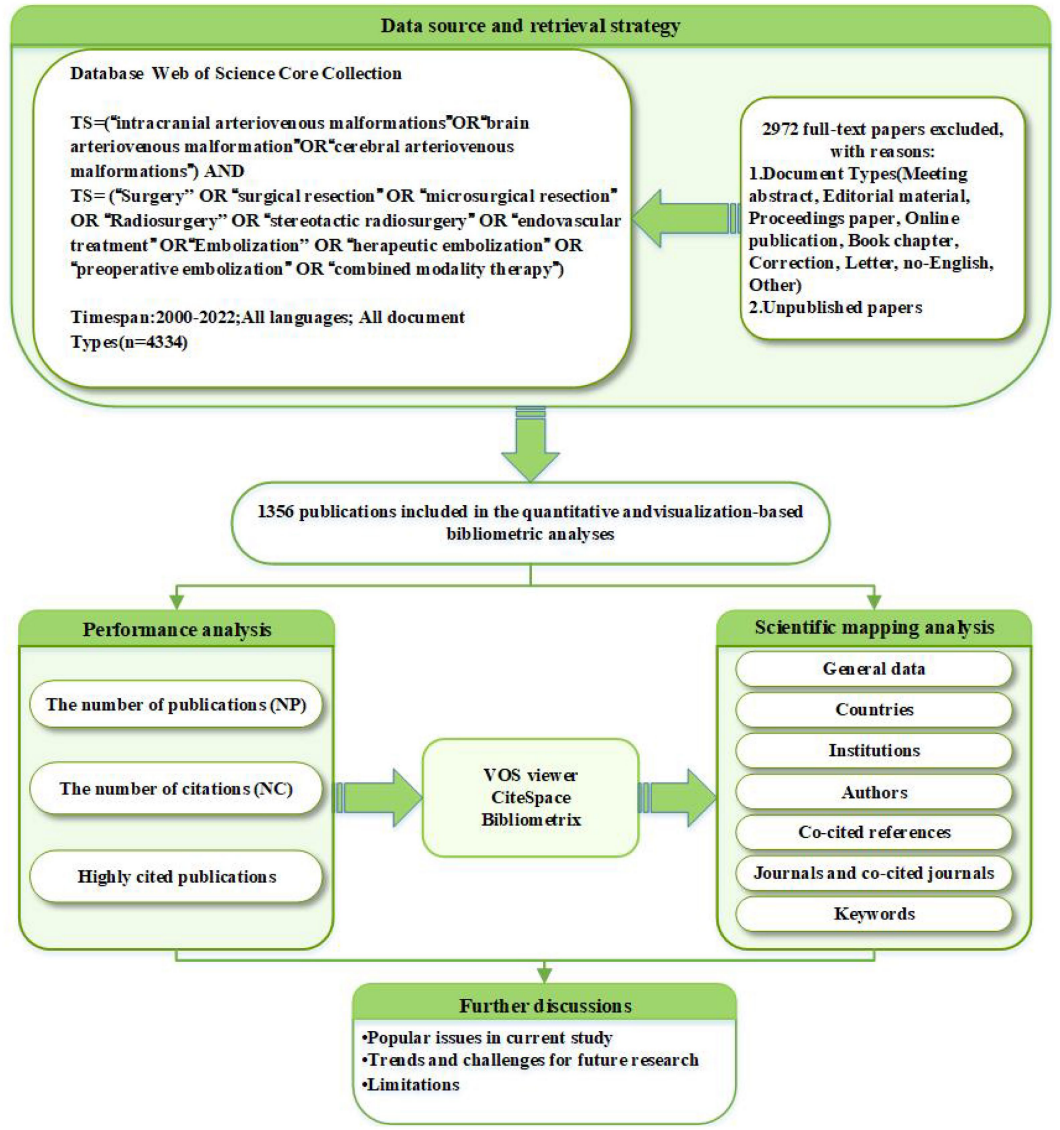
Flowchart for the research’s search process.

This bibliometric analysis selected the Web of Science Core Collection (WoSCC) as the primary database for data retrieval. In comparison with Scopus, Derwent, Google Scholar, the China National Knowledge Infrastructure (CNKI), and other renowned databases, the WoSCC encompasses a wider range of scientific publications and stands as the most frequently utilized database for bibliometric studies. The detailed data collection strategy is illustrated in Figure 1. A total of 1,356 English-language articles published between 2000 and 2022 were screened. The search strategy was presented as follows: TS = [(“intracranial arteriovenous malformations” OR “brain arteriovenous malformation” OR “cerebral arteriovenous malformations”) AND (“surgery” OR “surgical resection” OR “microsurgical resection” OR “radiosurgery” OR “stereotactic radiosurgery” OR “endovascular treatment” OR “embolization” OR “therapeutic embolization” OR “preoperative embolization” OR “combined modality therapy”)]. Retrieved papers were saved in a plain text format and exported as complete text citation records, named “download.txt.”

### 2.2 Data analysis

To import and analyze the bibliometric data from the WoS Core Collection, we employed various platforms and tools: R software (version 4.2.2), the Bibliometrix package (version 3.1) (16), VOSviewer (version 1.6.16) (17), CiteSpace (version 6.2.R2) (18), and Origin2021. These tools are frequently used to facilitate the visualization of critical bibliometric indicators such as co-authorship, co-occurrence, and co-citation analyses. Co-authorship analysis focuses on the collaborative networks among different institutions, authors, and countries, revealing patterns of scholarly collaboration. Co-occurrence analysis quantifies the relationships among various research elements such as keywords and themes, providing insights into the intrinsic connections within a research field. Co-citation analysis employs statistical methods to highlight the frequency and relationships of academic paper citations, mapping the structure of knowledge and academic influence. This study compiled foundational data, encompassing authors, institutions, countries/regions, journals, keywords, and references, to support subsequent analyses.

CiteSpace is a widely used tool in research fields for the visualization analysis of scientific literature trends, patterns, and the evolution of knowledge, offering distinctive insights into the chronological development of citation data. It generates knowledge maps incorporating elements such as institutions, authors, countries, and citations; the connections between nodes reveal collaborative or co-citation relationships, with node size indicating frequency or significance and color gradients distinguishing different time periods (darker shades represent earlier years, lighter shades indicate recent years). Purple rings signify nodal centrality, indicating pivotal developments within a field. Utilizing CiteSpace, we performed citation and journal analyses, identifying high-frequency citation patterns among literature keywords to trace emerging citations and key terms. Journal co-citation maps reveal inter-journal citation relationships, and keyword timelines illustrate research topic progression.

VOSviewer offers standardized visualization solutions for various datasets based on probabilistic analysis. Compared to traditional bibliometric tools, it places a greater emphasis on the presentation of visual graphics. Within the software, nodes represent specific dimensions such as countries, journals, or keywords. The size of a node is determined by weighted metrics such as publication counts, frequencies, or citation counts, while color clustering denotes group affiliation. Connections between nodes are represented by lines, and the Total Link Strength (TLS) index quantifies the intensity of collaboration or co-citation between them. This study leveraged VOSviewer for the visual analysis of linkages between countries, institutions, and authors.

The Bibliometrix package within the R software environment was used to analyze co-authorship and publication data by region and country. Data tables were populated with metrics, including the impact factor (IF) and the Hirsch index (H-index), to enable a comprehensive interpretation of scientometric outcomes. The H-index is a metric used to measure the academic influence of a scholar or journal. It is defined as the number of papers published by a scholar that have been cited at least ‘H’ times.

## 3 Results

### 3.1 Global trend in publication outputs and citation

**Figure 2:**
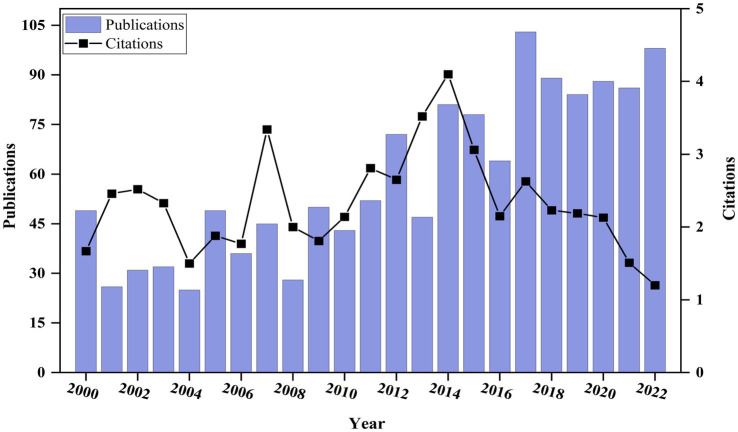
Global publication output and citation trend on bAVM research.

Since 2000, the number of publications in the field of bAVM has increased from 49 to 98 in 2022, showing a gradual upward trend in annual publication count (Figure 2). Furthermore, there were two peaks observed in the average citation count of articles, occurring in 2007 and 2014. The wave-like pattern suggests the potential for future peaks in this index. These findings indicate that the treatment of bAVM is a continuously evolving research field that merits ongoing attention.

### 3.2 Countries, institutions, and authors

#### 3.2.1 Countries

**Figure 3:**
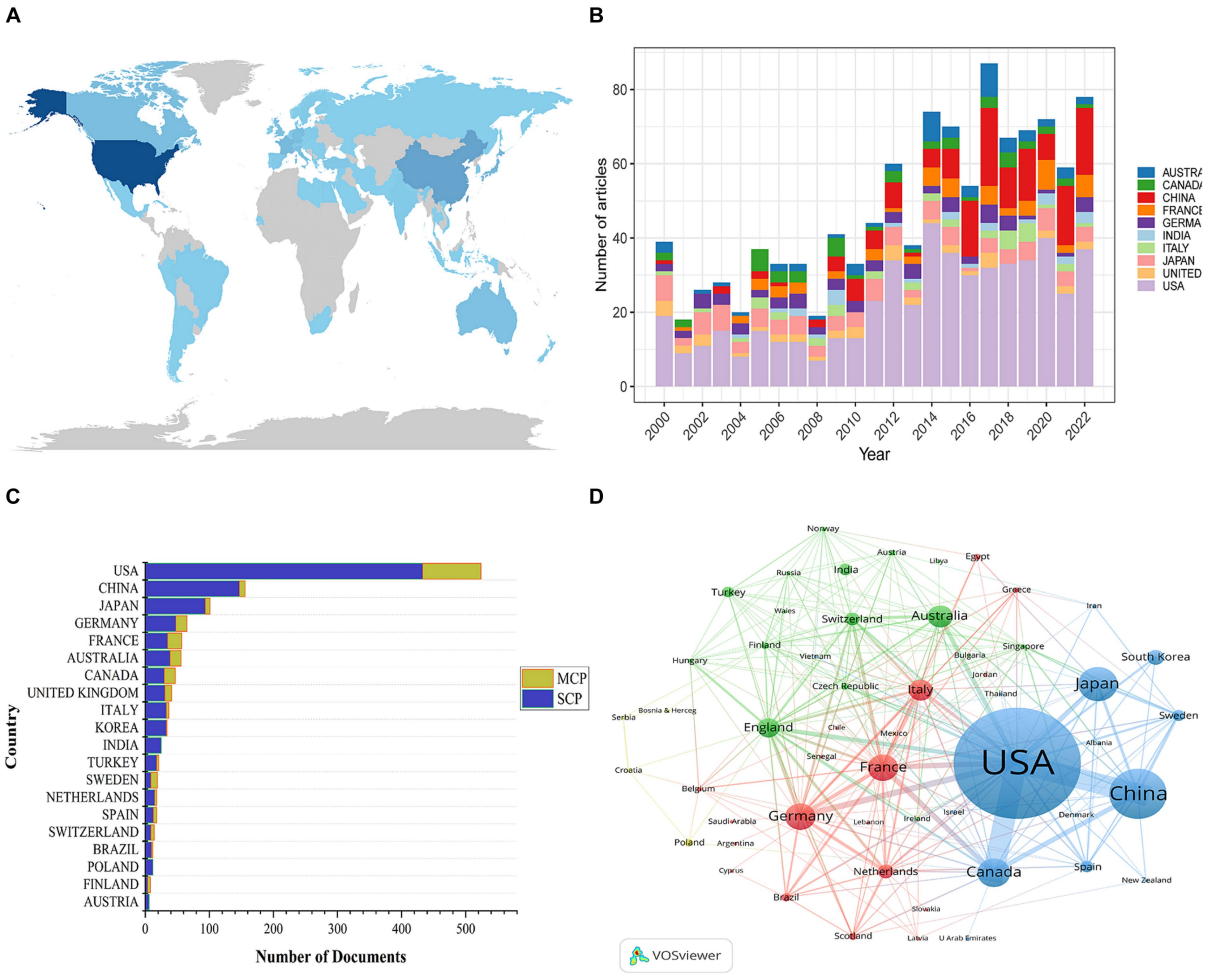
**(A)** Geographical distribution map based on the total publications of different countries. **(B)** The changing trend of the annual publication quantity in the top ten countries. **(C)** The number of documents published in different country and collaborative situation of corresponding authors. **(D)** The countries’ citation network visualization map was generated by using a VOSviewer.

A total of 64 different countries have published publications related to bAVM. According to the geographical distribution on the global productivity map, the relevant papers are primarily published in Asian, North American, and European countries (Figure 3A). Table 1 lists the top 10 countries with the largest number of total citations, and Figure 3B shows the annual publication counts for these countries throughout the period 2000–2022. China exhibits the highest annual growth rate in publications. The United States has the highest number of publications (524), followed by China (146) and Japan (101). The United States also has the highest citation count (15,699), with Japan ranking second (2,044). These data further affirm the proactive influence and significance of the United States in the field of bAVM treatment. In Figure 3C, SCP represents the number of publications co-authored by authors of the same nationality, while MCP represents the number of publications co-authored with authors from other countries. The United States ranks first in both SCP and MCP. In Figure 3D, a global collaboration analysis of the 64 countries was conducted using VOSviewer, where the thickness of the lines connecting countries represents the level of collaboration between them. Based on the thickness of the lines, it appears that the United States, China, and Canada are the core countries in this network. Notably, the collaboration between the United States and China reached 47 times, which is the highest among all collaborations.

**Table 1:** The top 10 countries with the largest number of total citations.

Rank	Country	TC	Average article citations	NP
1	USA	15,699	30.00	524
2	JAPAN	2044	20.20	101
3	FRANCE	1883	33.00	57
4	CANADA	1732	36.10	48
5	GERMANY	1,583	24.40	65
6	CHINA	1,231	8.40	146
7	AUSTRALIA	1,031	18.40	56
8	NETHERLANDS	994	55.20	18
9	UNITED KINGDOM	798	19.50	41
10	SWEDEN	670	35.30	19

#### 3.2.2 Institutions

**Figure 4:**
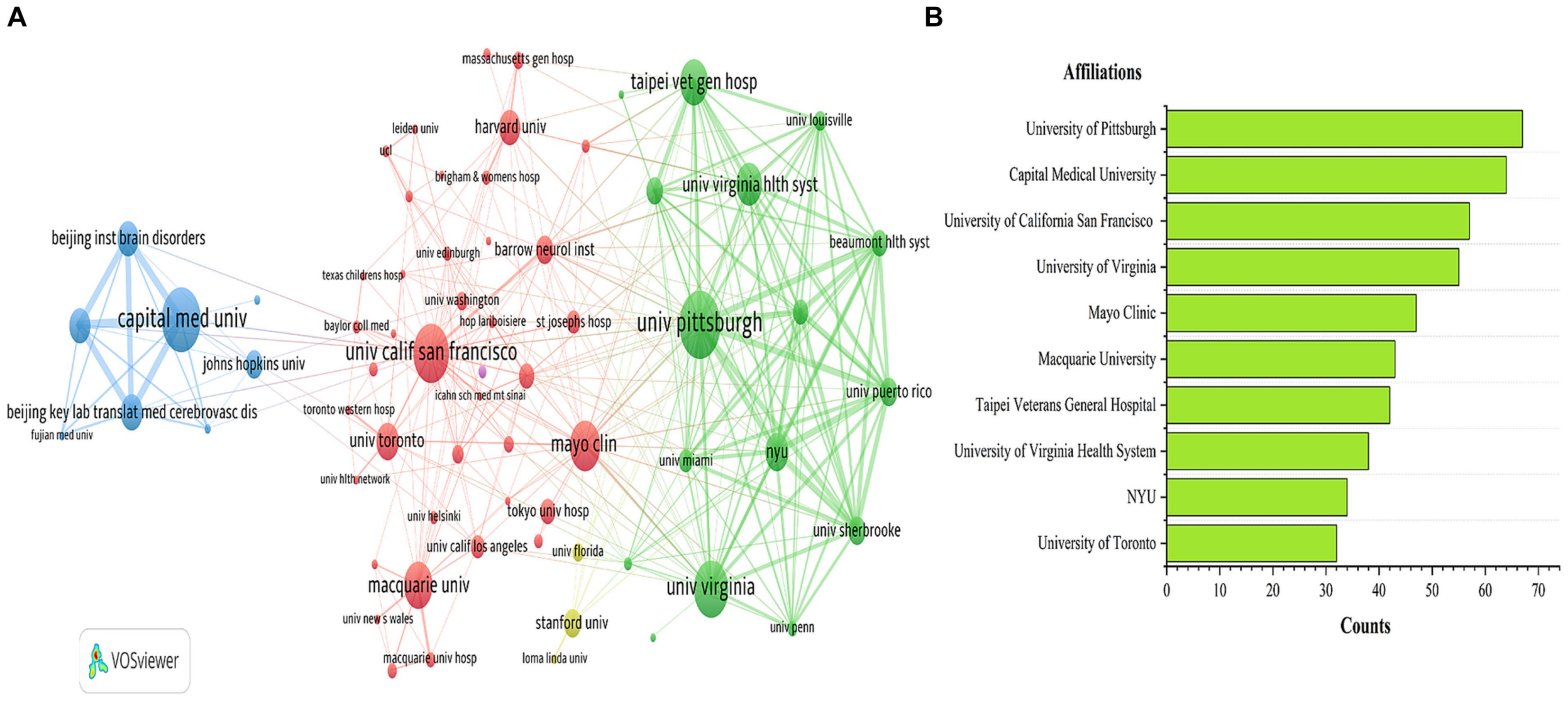
Affiliation collaboration network **(A)** and top 10 affiliations **(B)** in the field of bAVM.

The network visualization map of institutional collaboration was generated using VOSviewer. Institutions with a publication count of ≥6 were visually presented. Figure 4 illustrates the network of institutional collaboration and the top 10 institutions based on publication count. This figure includes 75 nodes and 309 links. The top three institutions with the highest TLS (Total Link Strength) are University of Pittsburgh (TLS = 211), NYU (TLS = 186), and University of Puerto Rico (TLS = 180). University of Pittsburgh has published 67 papers, while Capital Medical University and University of California San Francisco have published 64 and 54 papers, respectively. It is worth noting that the majority of the top 10 institutions are located in the United States and China. However, the research between institutions is relatively independent, mainly demonstrating collaboration within their respective countries.

#### 3.2.3 Authors

**Figure 5:**
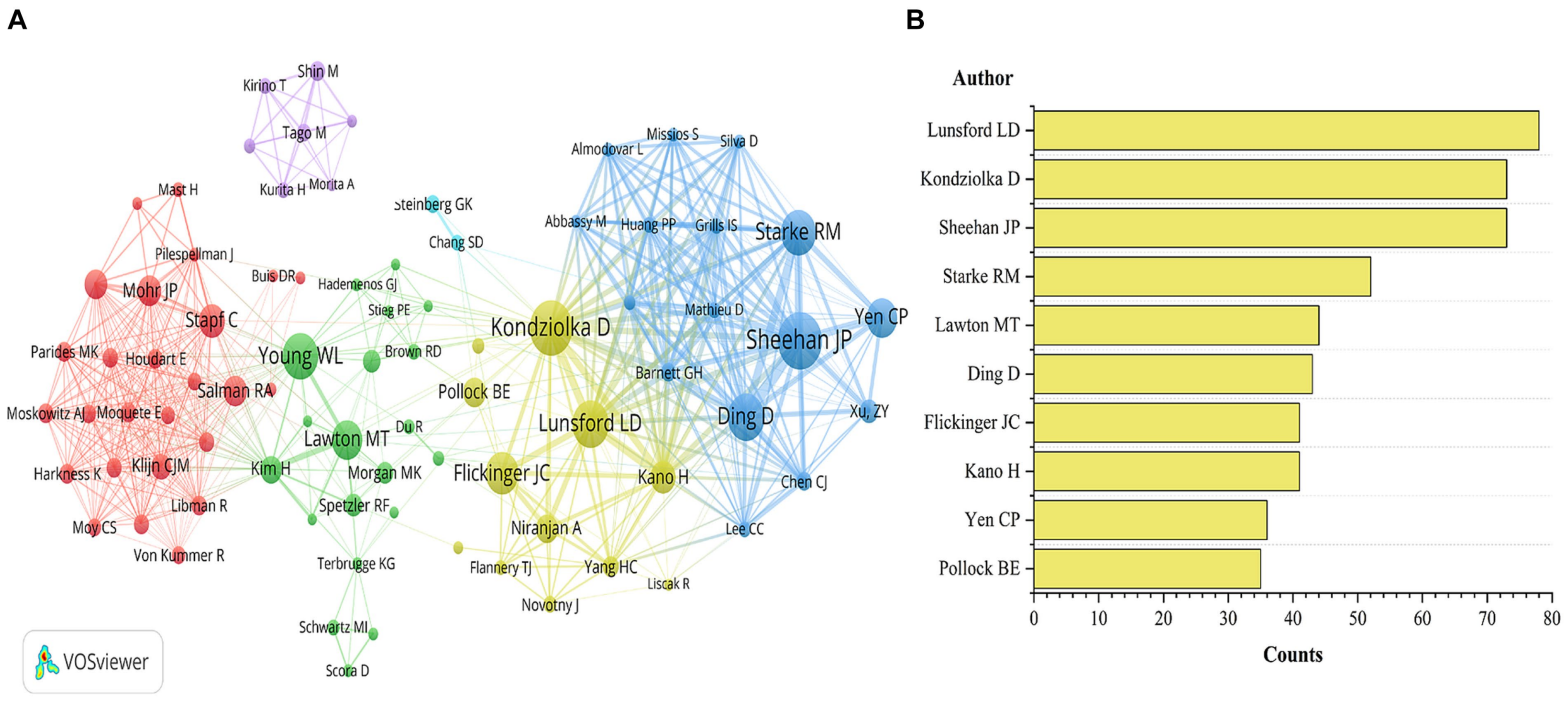
Author collaboration network **(A)** and top 10 authors **(B)** in the field of bAVM.

Figure 5 shows the author’s co-occurrence map and the top 10 authors of the publication. Lunsford LD, Kondziolka D, and Sheehan JP are the top three authors in terms of publication volume, affiliated with the Department of Neurosurgery at the University of Virginia, the Department of Neurosurgery at the University of Miami, and the Department of Neurosurgery at the University of Pittsburgh, respectively. Table 2 presents the top 10 authors according to TC rankings. These authors are highly respected experts in the field of bAVM research. Their studies have provided important scientific foundations for the diagnosis and treatment of bAVM, with positive implications for patient prognosis and quality of life.

**Table 2:** The top 10 authors with the largest number of total citations.

Rank	Author	TC	H-index	NP
1	Kondziolka D	3,006	29	51
2	Sheehan JP	2,827	33	73
3	Lunsford LD	2,429	27	54
4	Young WL	2,367	19	25
5	Starke RM	2,281	32	52
6	Flickinger JC	2034	20	23
7	Lawton MT	1951	22	43
8	Yen CP	1933	29	36
9	Ding D	1822	28	43
10	Stapf C	1,541	12	13

With the assistance of VOSviewer software, we identified 83 authors who have been cited more than 400 times and analyzed their co-occurrence patterns, revealing the collaborative network among key figures in this field (Figure 5). The researchers’ collaboration network is decentralized, primarily composed of three larger clusters. Kondziolka D, Sheehan JP, and Lunsford LD form the largest and most influential cluster, with Kondziolka D and others primarily focusing on the study of complications and risks associated with radiosurgery treatment for bAVM. Young WL and colleagues primarily investigate the genetic, molecular, cellular, and developmental mechanisms underlying cerebral arteriovenous malformation etiology. Stapf C and Mohr JP’s research cluster focuses on evaluating the effectiveness and safety of bAVM treatments through randomized controlled trials, which yield crucial evidence for guiding clinical practice and treatment decisions.

### 3.3 Analysis of journals and co-cited journals

As shown in Table 3, the most frequently cited journal is the Journal of Neurosurgery (6,182 citations), followed by Neurosurgery (6,024 citations) and Stroke (2,336 citations). Furthermore, the H-index (47 and 45) and the number of published papers (143 and 157) in the Journal of Neurosurgery and Neurosurgery, respectively, are significantly higher than those of other journals, indicating their outstanding contributions to bAVM research. Additionally, a small number of articles on bAVM have been published in top-tier journals such as Lancet and New England Journal of Medicine.

**Table 3:** The top 10 sources with the largest number of total citations.

Rank	Sources	TC	H-index	NP	IF
1	Journal of Neurosurgery	6,182	47	143	4.6
2	Neurosurgery	6,024	45	157	5
3	Stroke	2,336	23	36	8.8
4	American Journal of Neuroradiology	1,549	19	34	4.1
5	International Journal of Radiation Oncology Biology Physics	1,152	17	28	6.6
6	World Neurosurgery	1,140	18	129	2.1
7	Lancet	975	2	2	118.1
8	Acta Neurochirurgica	804	16	46	2.4
9	Journal of Clinical Neuroscience	720	16	54	2.1
10	Journal of Neurosurgery-Pediatrics	716	16	31	1.9

**Figure 6:**
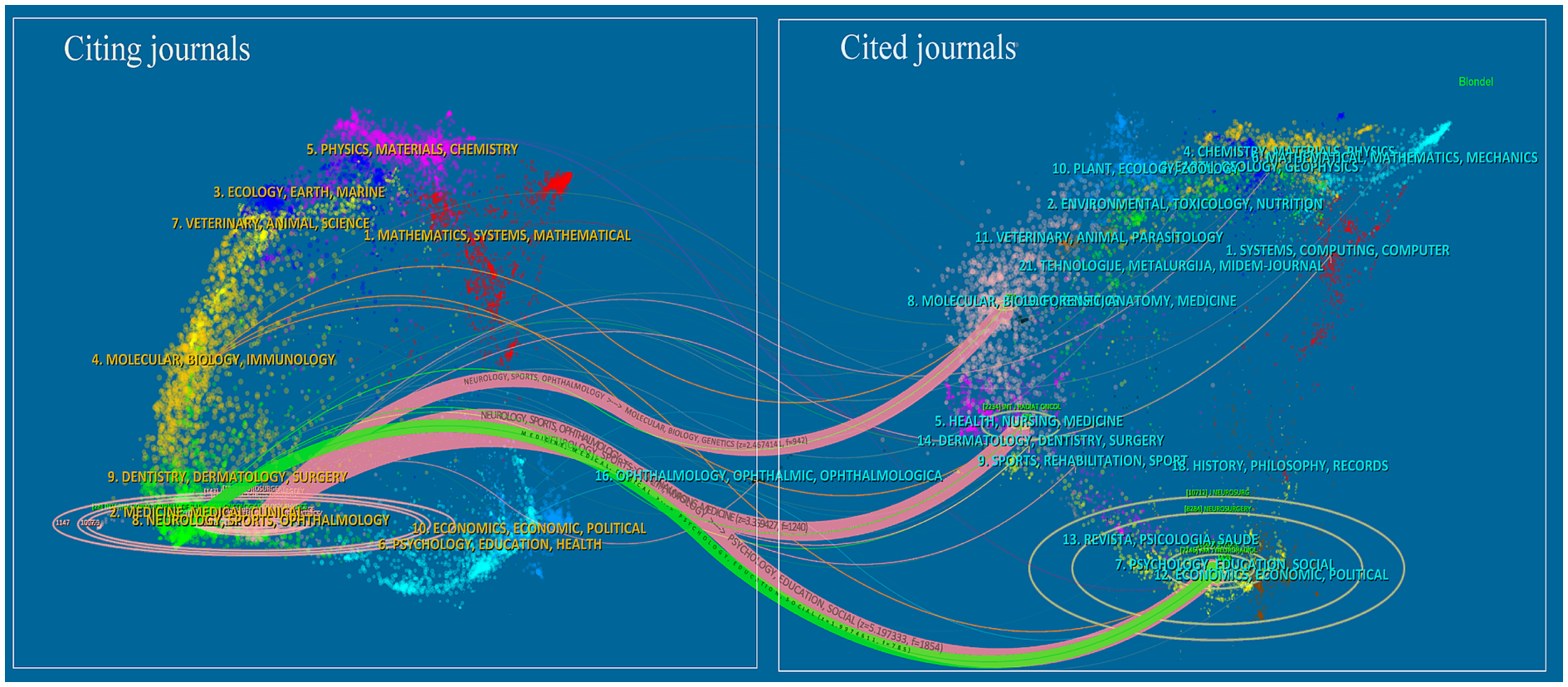
Dual-map overlay of the journals on bAVM research.

The collection of past literature, represented by the cited references, serves as the foundation and evidence for the forefront of disciplinary research. Figure 6 depicts the overlay of journal networks in the field of bAVM treatment from 2000 to 2022. Citing journals are shown on the left, cited journals on the right, and colored paths indicate citation relationships. The width of the connected paths is closely related to the frequency of citations on the z-score scale. It can be seen that there are three citation paths, suggesting that the birth of this 1 topic on the left required the knowledge base provided by these three different topics on the right. This means that studies published in neurology/sports/ophthalmology journals tend to cite studies published in molecular/biology/genetics/health/nursing/medicine/psychology/education/social journals.

### 3.4 Analysis of top co-cited references and the top 10 most locally cited articles

The top 10 most cited publications in the local area are shown in Table 4. The first and second ranked publications were authored by Stapf C et al., with 271 and 195 local citations, respectively. Firstly, Stapf C et al. proposed in 2006 that hemorrhagic bAVM presentation, age, deep bAVM location, and isolated deep venous drainage were independent predictive factors for intracranial hemorrhage during follow-up (3). Secondly, they designed the first ARUBA trial in 2014 to compare the effects of drug treatment and interventional treatment (19). Although the trial faced strong criticism in various aspects, it undeniably provided important references for the observation and treatment of unruptured brain arteriovenous malformations. The other publications mainly focused on the natural history of bAVM, treatment modalities, risk determination, and assessment, making remarkable contributions to the field of bAVM research and promoting the understanding of bAVM natural history and the development of treatment approaches.

**Table 4:** The top 10 most locally cited articles.

Document title	Corresponding author	Journal	Local citations	IF
Medical management with or without interventional therapy for unruptured brain arteriovenous malformations (ARUBA): a multicentre, non-blinded, randomised trial	Stapf C	Lancet	271	118.1
Predictors of hemorrhage in patients with untreated brain arteriovenous malformation	Stapf C	Neurology	195	10.3
Natural history of cerebral arteriovenous malformations: a meta-analysis	Du Rose	Journal of Neurosurgery	150	4.6
A proposed radiosurgery-based grading system for arteriovenous malformations	Pollock BE	Journal of Neurosurgery	149	4.6
Treatment of brain arteriovenous malformations: a systematic review and meta-analysis	Van Beijnum J	JAMA	148	120.7
Natural history of brain arteriovenous malformations: a long-term follow-up study of risk of hemorrhage in 238 patients	Laakso A	Neurosurgery	145	4.8
A supplementary grading scale for selecting patients with brain arteriovenous malformations for surgery	Lawton MT	Neurosurgery	142	4.8
The risk of hemorrhage after radiosurgery for cerebral arteriovenous malformations	Maruyama K	New England Journal of Medicine	128	158.5
The natural history and predictive features of hemorrhage from brain arteriovenous malformations	Tymianski M	Stroke	122	8.3
Recommendations for the management of intracranial arteriovenous malformations: a statement for healthcare professionals from a special writing group of the Stroke Council, American Stroke Association	Ogilvy CS	Circulation	118	37.8

**Figure 7:**
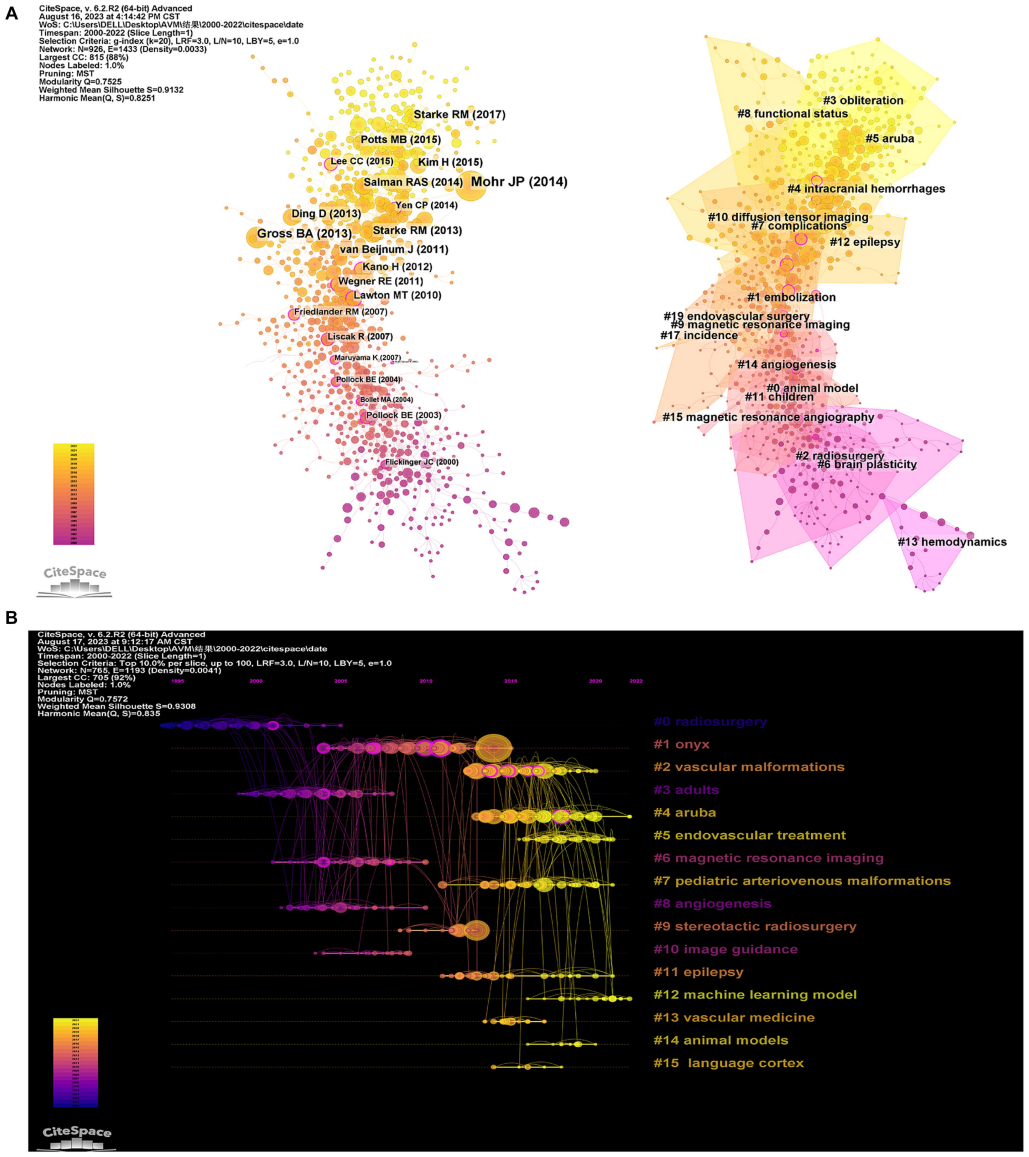
Citespace visualization map of Cluster view **(A)** and timeline view **(B)** of co-citation references. The time evolution is indicated with different colored lines, and the nodes on the lines indicate the references cited.

Figure 7A shows a graphic representation of the reference cocitation network study. The results indicate a modularity Q value of 0.7525 (>0.3) and an average silhouette S value of 0.9132, suggesting that the clustering is reasonable and feasible. Using CiteSpace, a total of 17,811 co-cited references were analyzed, with the top 10% most cited references selected as the selected group to display the cocitation relationships (Figure 7B). The co-cited references are also presented in a timeline perspective, illustrating how research hotspots have evolved. Based on the clustering results, it can be divided into 20 clusters. The “animal model” (#0) is the largest cluster, while “hemodynamics” (#13) represents the initial research focus in the field.

**Figure 8:**
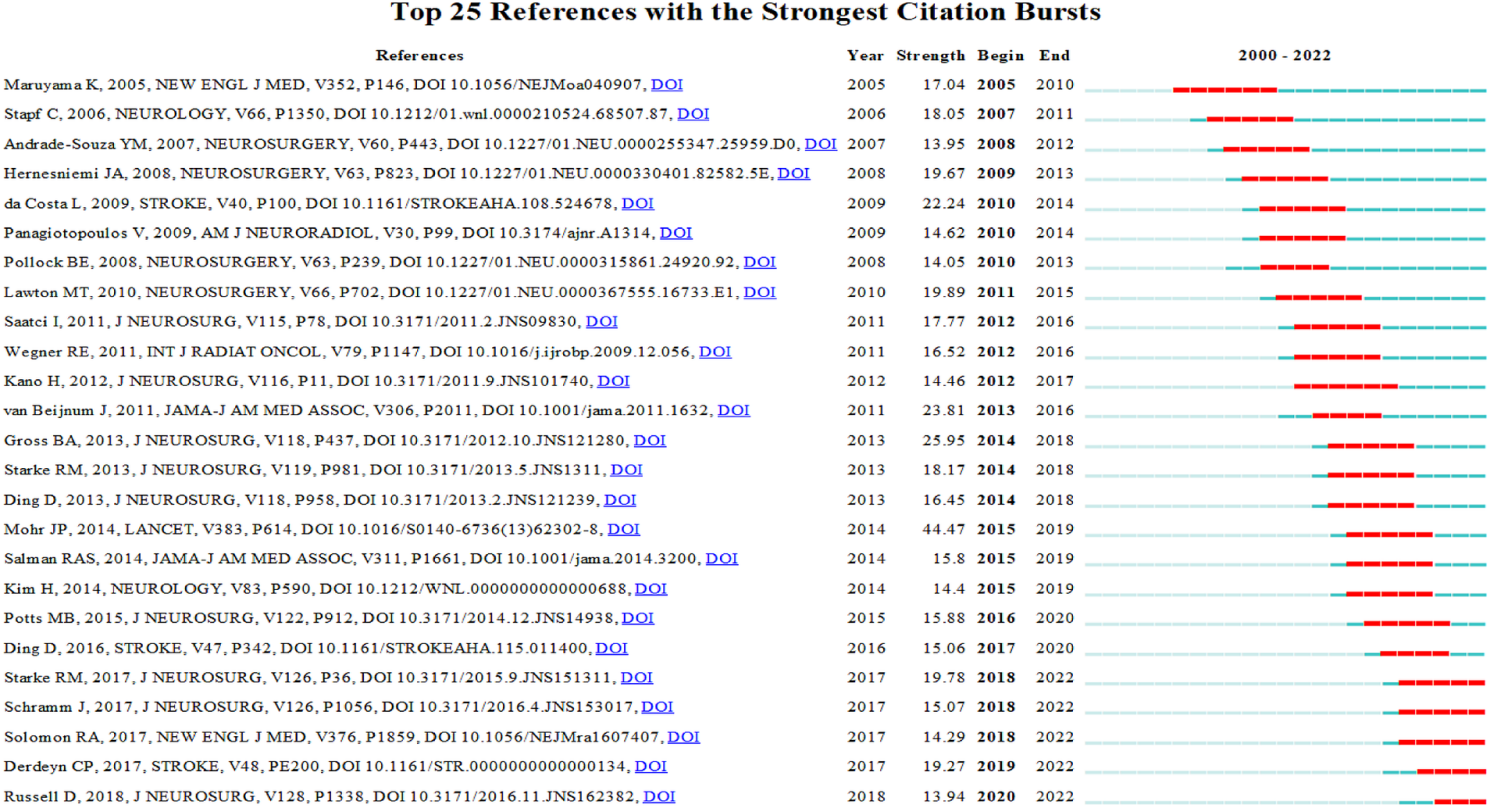
CiteSpace visualization map of top 25 references with the strongest citation bursts.

The current research hotspots, such as “aruba” (#4) and “machine learning model” (#12), indicate increasing attention from scientists towards bAVM treatment. Figure 8 displays the top 25 bursts referenced papers. The citation count in this field has been increasing since 2005, with many co-cited references remaining widely cited in the following years, indicating that bAVM treatment continues to be a topic of ongoing research interest.

### 3.5 Keywords analysis of research hotspots

Keyword co-occurrence analysis provides valuable insights into areas of interest and future research directions within discipline. These keywords represent the main topics covered in the publications, and a total of 837 keywords met the threshold for retrieval from titles, abstracts, and keywords. High centrality keywords indicate the status and influence of corresponding research content in the field, while high-frequency keywords represent current hot topics. Table 5 displays the high-frequency keywords found in relevant publications after excluding search terms and meaningless words. These include “intracerebral hemorrhage,” “gamma knife radiosurgery,” “medical management,” “natural history,” and “risk.” additionally, high-centrality keywords such as “ct angiography,” “animal model,” “classification,” “brain stem,” and “cerebral blood flow” are identified.

**Table 5:** The top 10 most frequent and centralized keywords related to bAVM.

Rank	Keyword	Counts	Rank	Keyword	Centrality
1	Cerebral arteriovenous malformations	1,078	1	CT angiography	0.21
2	Stereotactic radiosurgery	492	2	Animal model	0.17
3	Brain	469	3	Classification	0.14
4	Intracranial hemorrhage	449	4	Magnetic resonance angiography	0.11
5	Gamma knife radiosurgery	431	5	Dose–response	0.1
6	Medical management	398	6	Angiography	0.09
7	Natural history	387	7	Aneurysms	0.09
8	Surgery	364	8	Adults	0.09
9	Endovascular embolization	330	9	Brain stem	0.09
10	Risk	289	10	Cerebral blood flow	0.08

**Figure 9:**
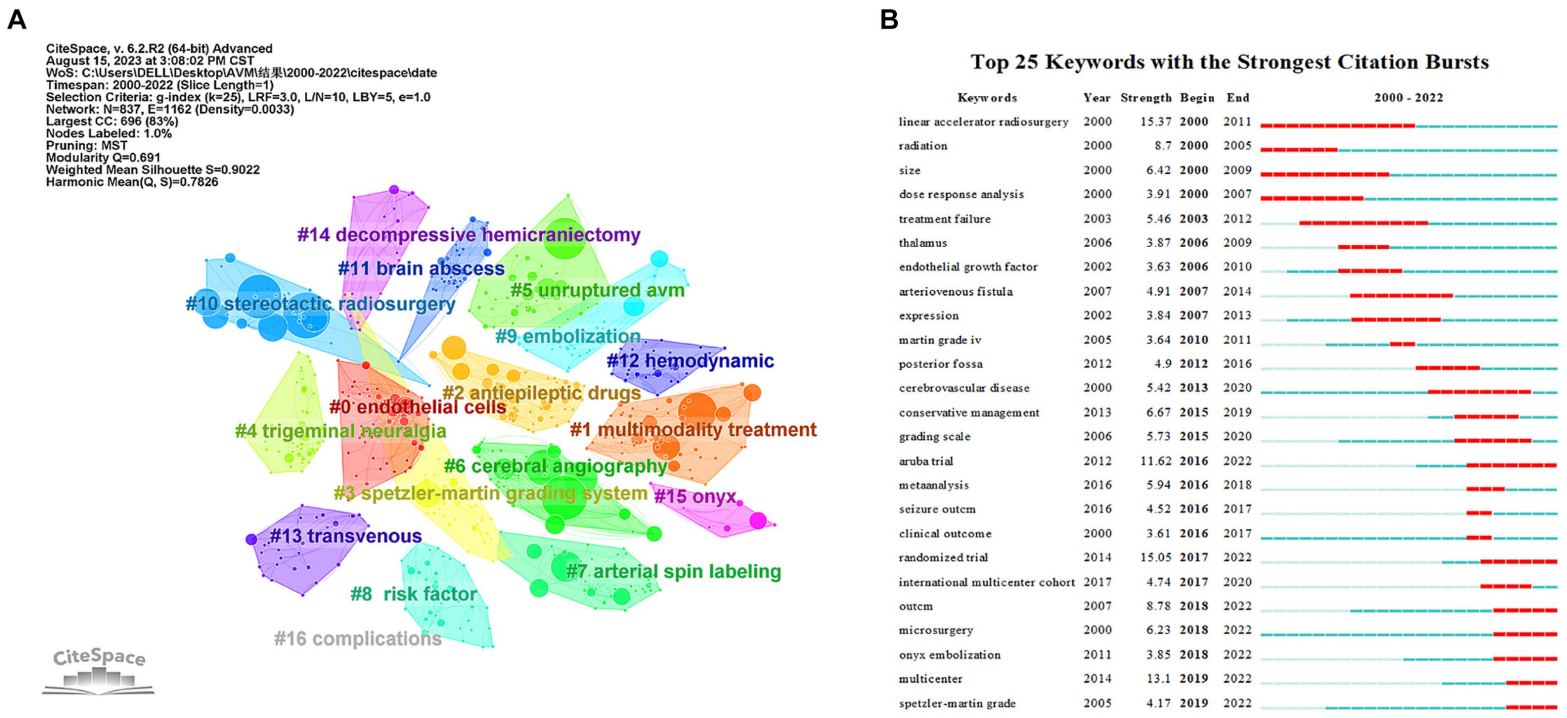
Analysis of Keyword Co-Occurrence. **(A)** Clustering co-occurrence map of the predominant keywords of studies. **(B)** Top 25 keywords with the strongest citation bursts.

Cluster analysis is a data mining technique used to identify and analyze important terms contexts trends and their relevance in a research area. Cluster analysis was performed on 837 keywords based on the log-likelihood ratio (LLR) of the keywords in Citespace. The clustering map was evaluated by the clustering modulus value Q and the clustering profile index S. It is generally accepted that Q > 0.3 means that the clustering structure is significant S > 0.5 means that the clustering is justified and S > 0.7 means that the clustering is convincing. In our study *Q* = 0.691 > 0.3 and *S* = 0.9022 > 0.7 which indicates that the keyword clustering structure is clear and the clustering is reasonable and reliable. The LLR calculation of the keywords resulted in 17 natural clusters. Figure 9A shows keyword clustering. Category 1: pathogenesis (#0 endothelial cells and #12 hemodynamic); the second category: imaging diagnosis and evaluation (#6 cerebral angiography, #7 arterial spin labeling); the third category: treatment method (#1 multimodality treatment, #2 antiepileptic drugs, #5 unruptured avm, #9 embolization, #10 stereotactic radiosurgery, #13 transvenous, #14 decompressive hemicraniectomy, #15 onyx); the fourth category: risk prediction (#3 Spetzler-Martin grading system, #4 trigeminal neuralgia, #8 risk factor, #11 brain abscess, #16 complications).

Another key indicator of research frontiers, hotspots, and emerging trends is the intensity of keyword bursts (Figure 9B). Burst keywords with high intensity include “aruba trial” (2016–2022), “randomized trial” (2017–2022), “microsurgery” (2018–2022), “onyx embolization” (2018–2022), and “spetzler-martin grade” (2019–2022) continues until 2022, demonstrating that certain study fields have recently received a lot of attention.

## 4 Discussion

### 4.1 Global trends in research on the treatment of cerebral arteriovenous malformations

To comprehend the research findings of the global scientific community regarding the treatment of bAVM, we analyzed the current status, research, and publication trends of bAVM. Data from 2000 to 2022 were utilized, and a comprehensive analysis was performed using the R software in conjunction with the “Bibliometrix” package, as well as Citespace and VOSviewer software for identifying hotspots and publication trends. The analysis of annual output revealed a consistent increase in the number of publications over time. Regarding the average article citations per year, two notable peaks were observed, possibly attributed to the publication of locally influential articles. The peak in 2014 may be attributed to the publication of ARUBA. By examining the number of publications and citations, valuable insights can be gained into the scientific production pattern concerning bAVM.

According to the analysis of countries, the United States ranks first in terms of the number of publications and total citations in the field of bAVM-related research, demonstrating its significant productivity and influence. However, in comparison to the United States, China ranks second in the number of publications but exhibits significantly lower total citations. These findings suggest that Chinese scholars should allocate more effort towards improving research quality. The majority of the top ten most productive institutions are located in the United States, further affirming the country’s exceptional research standards. The top three prolific authors, namely Kondziolka D, Sheehan JP, and Lunsford LD, all from the United States, focus on comparing the effectiveness and safety of various treatment methods for cerebral arteriovenous malformations, as well as evaluating treatment outcomes and potential complications. Notably, Lunsford LD has made significant contributions to the development and refinement of Gamma Knife radiosurgery for bAVM treatment. Their research has significantly enhanced the understanding and management of this complex vascular disease.

Journals allow researchers to access information to choose the most suitable journal for publishing their articles. Journal of Neurosurgery, Neurosurgery, and World Neurosurgery are possibly the main journals for publishing articles on the treatment of bAVM. Additional articles can be submitted to these publications. Among the top 10 journals in the TC ranking, only Lancet has an impact factor higher than 10.0, but it has only published two articles. Lastly, it is worth noting that the top 2 journals (Journal of Neurosurgery and Neurosurgery) strike a balance between the quantity and quality of bAVM research, so focusing on these two journals will help us stay updated on the forefront of bAVM research.

### 4.2 Hotspots and emerging frontiers in bAVM treatment research

Prior bibliometric results have uncovered several focal issues within the field. Results from dual-map overlays suggest that the corpus of research on bAVM treatment is a product of multidisciplinary cross-fertilization, which may foster the development of new theories, technologies, or comprehensive care models incorporating physical, genetic, psychological, and social aspects of patient health. Keyword analysis indicates research hotspots in bAVM treatment concentrate around terms such as “aruba trial,” “randomized trials,” “microsurgery,” “onyx embolization,” and “Spetzler-Martin grade.” Furthermore, clusters derived from a timeline review to some extent reflect the ubiquity of these phenomena. This section will further discuss bAVM treatment research by combining the aforementioned factors, including current principal research themes, salient issues, future trends, and challenges, as well as limitations.

#### 4.2.1 Pathogenesis

**Figure 10:**
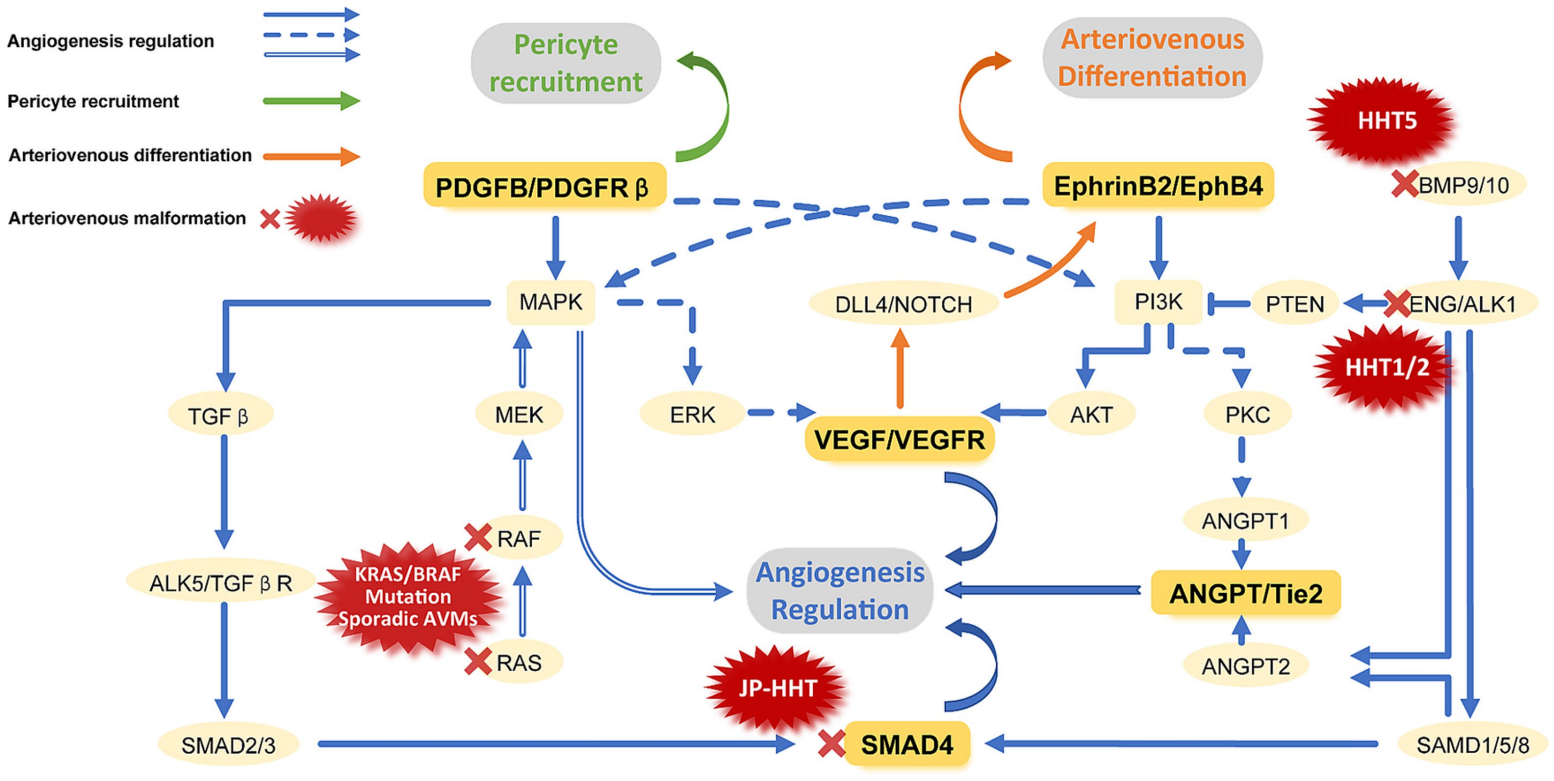
Signaling pathways involved in vascular development and causes of bAVM. The vascular development primarily includes three processes: regulation of Angiogenesis, recruitment of pericytes, and regulation of arteriovenous differentiation. Angiogenesis mainly involves the classic TGF-β signaling pathways and the PTEN-mediated non-TGF-β signaling pathway. Other potential pathways include ANGPT/Tie2, RAS, and VEGF mediated pathways. Pericyte recruitment is mainly mediated by PDGFB/PDGFR-β. Arteriovenous differentiation principally involves the differential expression of EphrinB2/EphB4 mediated by the DLL4/NOTCH pathway. Abnormalities in any pathway involved in vessel development may lead to the occurrence of bAVM. Familial bAVM mainly include HHT1, 2, 5 and JP-HHT, which are caused by mutations in ENG, ALK-1, BMP9 and Smad4, respectively. The Primary cause of sporadic bAVM is KRAS/BRAF mutations.

The etiology of bAVM is a central focus, but its pathogenesis remains uncertain and controversial. The bAVMs can be roughly divided into familial and sporadic (Figure 10). The Familial bAVM is related to the syndrome called hereditary hemorrhagic telangiectasia (HHT). Pathogenesis varies between subtypes of HHT, but they are primarily caused by inherited germline mutations, and all related to the transforming growth factor β (TGF-β) pathway (20). HHT type 1 was caused by loss-of-function mutations in one copy of the Endoglin (ENG) gene. The HHT2 type 2 can be attributed to the above-mentioned mutations in the activin-like kinase receptor 1 (ALK-1) gene (21). And mutations in SMAD4 cause JP (juvenile polyposis) – HHT (22, 23). These genetic mutations all function in endothelial cells (24–26). These inherited germline mutations, acting in conjunction with second hits including angiogenesis stimulation, responses to disturbance or injury, and abnormal blood flow, enhance PTEN phosphorylation and activate the PI3K/AKT pathway, thereby upregulating YAP/TAZ expression (27–32). Eventually, capillaries dilate abnormally and acquire vein markers. Sporadic bAVM mainly involves somatic mutations in RAS-MAPK–ERK pathway genes, including KRAS and BRAF mutations (33–35). Abnormal expression of platelet derived growth factor subunit B (PDGFB)/pdgf Receptor β (pdgfrβ) and angiopoietin (ANGPT)/Tie2 is associated with the recruitment of supporting structures (vascular pericytes and smooth muscle) during angiogenesis, which is the underlying mechanism of bAVM formation (36, 37). Crosstalk between multiple factors and the endothelial growth factor (VEGF)-Notch pathway can lead to disordered expression of arteriovenous specification-related genes, thereby promoting bAVM formation (38–40). This includes wall shear stress (WSS) modulating EFNB2 expression and endothelial-mesenchymal transition (EndMT) through the Notch pathway, and the effect of BMP/ALK1/SMAD signal transduction on Notch pathway (32, 41, 42). In addition, inflammatory responses involving macrophages, neutrophils, and lymphocytes affect angiogenesis and vascular remodeling, which are potential contributors to bAVM (32). For example, high WSS recruit macrophages to trigger inflammation by activating pro-inflammatory signaling (nuclear factor kappa-B: NF-κB; macrophage chemoattractant protein 1: MCP1; vascular cell adhesion molecule-1: VCAM-1) in ECs (43, 44). Macrophages can promote angiogenesis through the ANGPT-Tie pathway, and express high level of PDGFB to promote the recruitment of pericytes in new blood vessels, suggesting that macrophages may be an important mediator of bAVM vascular remodeling (45, 46). Studies targeting single nucleotide polymorphisms, microRNAs, differentially expressed genes, and single-cell omics all advance understanding of bAVM pathogenesis (Figures 1, 2).

#### 4.2.2 Imaging diagnosis and evaluation

Research in imaging diagnosis and evaluation is also a key focus in this field. Small or deep-seated bAVM may be difficult to accurately visualize on routine imaging examinations, which can increase the risk of missed or misdiagnosis. Therefore, the use of appropriate imaging studies is crucial for the diagnosis and guidance of treatment planning in such patients.

Firstly, digital subtraction angiography (DSA) is the reference standard for the evaluation and characterisation of bAVM, but conventional DSA still has difficulties in the diagnosis of complex and small lesions due to the rapid filling of blood and the effect of vessel overlap. Sandoval-Garcia et al. (47) used 2D-DSA, 3D-DSA, and 4D-DSA to analyze the normal blood vessels in a canine model, respectively. The results showed that 4D-DSA showed better display of normal vessels, and also predicted that the main advantage of 4D-DSA would be in displaying abnormal small vessels, especially in areas with overlapping vessels. Therefore, bAVM researchers have turned their attention to 4D-DSA, which has the advantage of observing lesions at any time, in addition to the advantage that 3D-DSA partially avoids the effect of vessel overlap. Recently Carolina SG et al. demonstrated the feasibility of 4D-DSA in the assessment of bAVM and that 4D-DSA provided better visualisation of bAVM vascular structures than conventional methods (48). Several other studies have also demonstrated the advantages of 4D-DSA over conventional DSA in terms of displaying the neurovascular anatomy of bAVM patients in more detail, being able to more clearly characterize the degree of stenosis of the draining veins, and also reflecting cerebrovascular hemodynamic characteristics (49–51). However, 4D-DSA is currently mainly used in the diagnosis of complex bAVM. Because 4D-DSA has the shortcomings of conventional DSA, the application of 4D-DSA in the diagnosis of bAVM is limited.

The advent of wide-detector CT has provided the groundwork for the utilization of 4D-CTA. In comparison to DSA, 4D-CTA offers cost-effectiveness and time efficiency over traditional DSA. Since the publication by Klingebiel et al. (52) reporting on the use of 4D-CTA in cerebrovascular diseases, it has gradually garnered attention in bAVM diagnostic research. Multiple studies have demonstrated that 4D-CTA possesses comparable or even superior capabilities to DSA in assessing the main feeding arteries, draining veins, and tumor nest size, with a higher detection rate for microcerebellar AVMs (diameter < 1 cm) compared to DSA (53, 54). Furthermore, it exhibits enhanced diagnostic accuracy in detecting cranial arteriovenous shunts (55). However, given the current technology’s imperfections and the limited scope of related research to small series and case reports, 4D-CTA is currently considered an adjunctive tool to conventional non-invasive diagnostic techniques.

The above concerns about radiation dose are no longer an issue when using MRA to evaluate bAVM. It uses both contrast-enhanced (CE) and non-contrast-enhanced (NCE, usually 3D TOF MRA) imaging techniques. CE-MRA offers advantages in visualising blood vessels, particularly in detecting slow flow, minimizing spin dephasing effects, and improving vessel delineation compared with TOF-MRA. To overcome the limitations of TOF-MRA and single-phase CE-MRA techniques in terms of temporal resolution while maintaining spatial resolution, four-dimensional CE-MRA has been used to assess bAVM. It has shown good agreement with DSA in assessing arterial blood supply, lesion size, and venous drainage, and is a reliable tool for predicting Spetzler-Martin grading and postoperative residual filling (56, 57), and may therefore provide another alternative examination for follow-up, especially in younger patients. 4D-MRA vs. DSA Although 4D-MRA and 4D-CTA have certain advantages over DSA, the temporal resolution is still inferior to that of DSA and the detection rate of draining veins is not high (58), the imaging time is longer than that of 4D-CTA, it is highly sensitive to motion artefacts and prone to image blurring, and there are many contraindications, which to some extent limit its use in the clinic.

Overall, the aforementioned novel techniques hold significant value and promise widespread application in bAVM diagnosis. As our comprehension of bAVM deepens and imaging technology develops, the utilization of diverse innovative techniques in bAVM diagnosis is poised to expand further.

#### 4.2.3 Treatment modalities

Therapeutic decisions for bAVM are currently of great interest, and research into treatment options has been a major focus of the field. Treatment options for bAVM include conservative treatment, endovascular treatment, stereotactic radiotherapy (SRS), microsurgery, and combination therapies. Although numerous treatment options exist, complete resection remains a challenge for the most experienced surgeons.

The keywords “aruba trial” and “randomized trial” have gained popularity due to the therapeutic dilemma of whether to intervene in unruptured bAVM and the controversy surrounding the results of the ARUBA trial. The emergence of the SAIVM prospective bAVM cohort study and the ARUBA study have raised questions about the rationality of intervention in unruptured bAVM (19, 59). These studies suggest that asymptomatic bAVM should be treated conservatively. Conservative treatment may be an option for older patients with underlying diseases, higher Spetzler-Martin grades, and lower risks of bAVM rupture and bleeding. However, it should not be considered a universal treatment for unruptured bAVM. The ARUBA trial results are flawed in multiple aspects of study design, progression, and analysis/conclusions. After ARUBA, several observational studies were conducted to assess the safety and effectiveness of interventional treatment methods. These studies indicate that good outcomes can be achieved with careful patient selection and application of treatment techniques. Despite the widespread controversy and concern over the results of ARUBA, intervention rates in the United States for the treatment of unruptured bAVM do not appear to have changed significantly. This may reflect the fact that physicians and patients in clinical practice still weigh the limitations of the ARUBA study while considering other evidence and experience to continue utilizing existing treatments.

The keyword “microsurgery” has emerged as a focal point, indicating both the recognition of microsurgery as the gold standard for the treatment of bAVM and the continued advancement of the technique. Microsurgery is a major therapeutic approach for bAVM, particularly for ruptured bAVM with acute hemorrhage requiring haematoma evacuation. Numerous studies have described the risks and benefits associated with microsurgical treatment of bAVM (60, 61). Compared to alternative therapies, the main advantages of microsurgery include a higher likelihood of complete lesion eradication, immediate elimination of bleeding risk, and durable long-term results. However, as an invasive procedure, it carries inherent risks such as prolonged recovery and the potential for perioperative neurological complications. To improve safety and efficacy, several adjunctive technologies have been integrated, including functional MRI and diffusion tensor imaging for fiber tractography, stereotactic neuro-navigation, endovascular embolization, and intraoperative angiography. In addition, the Spetzler-Martin grading system is routinely used to predict the complexity and potential risks of surgical resection. Surgical resection is the treatment of choice for patients with lower Spetzler-Martin Grade I-II bAVM. For more complex bAVM with higher Spetzler-Martin grades, the disparity in recognition and technical capabilities between medical centers fuels the ongoing debate about surgical intervention (62, 63). Certainly, as assistive technologies such as augmented reality (AR), virtual reality (VR), surgical simulators, and robotic surgery continue to advance, there is potential to enhance surgical planning and execution, improve the predictability and precision of microsurgical outcomes, and thereby enable successful treatment of the vast majority of complex bAVM.

Stereotactic radiotherapy is mainly indicated for bAVM that are <12 cm in size or < 3 cm in diameter, deep in location, and unsuitable for surgery, or as a complementary treatment for residual lesions after treatment by other methods (6). For large bAVM, stereotactic radiotherapy has a high rate of revascularisation, a low rate of occlusion of the malformed mass, and a risk of radiological nerve injury, resulting in a poor overall outcome (64). The advantages over other treatment options are that it is non-invasive, has minimal risk of acute complications and is performed as an outpatient procedure, with the main disadvantages being the lack of immediate cure and the potential for long-term adverse effects of radiation. Therefore, as with other forms of treatment, the indications for stereotactic radiotherapy for bAVM needs to be judged, and adequate evaluation is required for specific bAVM radiotherapy.

The keyword “onyx embolization” is prominent, reflecting the sustained interest and in-depth exploration by researchers in the field of endovascular treatment of bAVM. In recent decades, significant advancements have been made in embolic agents and microcatheter technology for bAVM therapy. Liquid embolic agents commonly used in clinical practice include cyanoacrylates, Onyx, Squid, PHIL, and their various subtypes. Cyanoacrylates were once widely used, but their frequency of application has declined with the advent of Onyx. Onyx is known for its robust scientific research and positive outcomes in the treatment of cerebral vascular malformations, which testify to its safety and efficacy. However, some drawbacks of Onyx have been reported, such as loss of visibility with prolonged injection time and artifact issues on cross-sectional imaging, such as CT scans. To overcome these limitations of Onyx, new embolic agents such as Squid and PHIL have been developed. These agents are characterized by their low viscosity and have shown promising potential, results, and safety profiles in preclinical and clinical studies. Previous case reports have shown some success in treating bAVM with PHIL and Squid. However, further extensive multicenter studies and randomized controlled trials are needed to comprehensively assess their safety and efficacy compared to Onyx.

Embolization is often used as part of multimodal treatment for bAVM. However, recent studies have explored embolization as a stand-alone therapeutic strategy for complete occlusion of bAVM, particularly in patients with Spetzler-Martin grades I-II (65), due to ongoing technological advancements. Embolization offers the advantage of reduced invasiveness and fewer surgical interventions (65–67). However, its main drawback is the complete occlusion rate, which does not exceed 51% with embolization alone. However, in highly selective patients, initial embolization can achieve complete occlusion rates exceeding 90%. Therefore, it may be more meaningful to consider intervention based on individual patient characteristics. It is important to note that this therapeutic approach has a significant complication rate, and the latest clinical guidelines for bAVM treatment do not recommend embolization as the definitive treatment strategy (6, 68). The transition of endovascular embolization from an adjunct to a definitive treatment modality remains controversial.

For complex bAVM with deep location, involvement of functional areas, and massive volume, single-modality treatment carries an extremely high risk, necessitating a multimodal approach. It is important to note that multimodal treatment may be the safest approach for certain bAVM, but it may also lead to the cumulative risks of various treatment modalities. Two ongoing studies may provide answers in the future (69, 70).

#### 4.2.4 Risk prediction and future directions

The “Spetzler-Martin grading” system has emerged as a prevalent keyword within the neurosurgical field, reflecting the ongoing efforts to devise a grading schema that is both efficient and accurate. Such systems are crucial for informed patient selection, as they are integral to minimizing the risk of disability and maximizing therapeutic outcomes. Numerous classifications have been developed, among which the Spetzler-Martin grading system is widely accepted for its simplicity and practicality. In response to the complications associated with categorizing Grade III AVMs within this framework, revisions and novel supplemental schemes have been proposed, including the Lawton-Young (LY) grading system and the supplemented-SM (Supp-SM) grade. Recently, scales dedicated to cerebellar AVMs, grading of AVM-related intracerebral hemorrhages for clinical outcome prediction, and ruptured arteriovenous malformation grading have been introduced (71–73). These systems have been influential in both surgical planning and prognosis prediction. However, traditional grading systems, such as the Spetzler-Martin system, may require further optimization, as modern imaging techniques play an increasingly vital role in the functional assessment of various brain regions, aiding in the minimization of postoperative neurological deficits. For instance, the traditional concept of brain eloquence zones may not be sufficiently accurate, as it overlooks critical areas such as the subcortical white matter. Additionally, there is no clear standard for defining the clinical significance of the distance between an AVM and adjacent critical brain regions. Furthermore, studies have indicated that areas previously considered “silent” may be functionally active in the brain with AVM. Jiao et al. have recently developed an exploratory new scoring system that addresses the aforementioned concerns. In their proposed HDVL model, a retrospective analysis of 201 patients demonstrated that the HDVL scoring system outperforms traditional grading systems in predictive capability (74). However, further clinical trials are necessary to validate the actual benefits of this model. Additionally, the increasing trend of utilizing combination therapies for bAVM management introduces complexity, necessitating more comprehensive classification schemes to accurately predict the outcomes of such combined treatments. Machine learning models may offer a promising direction, with some studies already showing successful applications (75–77). Moreover, as research into bAVM at cellular and molecular levels progresses, an increasing number of genetic, molecular, and histological predictors are being recognized as risk factors for bAVM hemorrhage, suggesting future classification schemes might also need to incorporate these elements. Consequently, future directions aim to refine current methods, integrate new diagnostic tools, and employ data-driven approaches to optimize patient prognosis and personalized care.

In addition to the aforementioned strategies, another crucial direction is the desire to minimize invasive interventions through pharmacotherapy. Currently, the development of pharmacological treatments is relatively slow, with research primarily focusing on inhibiting the progression of AVMs, inducing abnormal vascular occlusion, and stabilizing vessels. Zhu et al. were the pioneers in demonstrating, with a murine model, that both sirolimus and lenalidomide suppress the development of bAVM and improve the vasculature integrity of existing bAVM (78). Walker et al. observed that bevacizumab treatment reduced vascular density and the prevalence of dysplastic vessels in a bAVM mouse model (79). The findings of Hashimoto et al. suggest that doxycycline holds the potential in reducing MMP-9 activity within AVM tissues and stabilize vessels prone to rupture (80). However, to date, only bevacizumab has progressed to clinical trials in humans, while other approaches remain in a transitional phase from early laboratory research to clinical application. The clinical advancement of these pharmacotherapies would represent a significant breakthrough. Moreover, the field might benefit from a multifaceted approach where drugs are used in concert with conventional treatments, such as surgery or modulate bAVM pre- or post-operatively, to minimize risks. Another consideration is the need for customized treatment strategies, tailored to the individual characteristics of each bAVM, including size, location, and genetic composition, which may impact the efficacy and safety of a given drug. In conclusion, while pharmacotherapy for bAVM holds considerable promise, extensive research is still necessary to fully understand the optimal use, long-term efficacy, optimal dosages, and integration of these drugs into the current treatment paradigms for bAVM.

The present study examines the developmental trends and potential frontiers in the research on the treatment of cerebral arteriovenous malformations from a bibliometric perspective for the first time. However, the study has several limitations. Firstly, only English articles/reviews were included in the search, and non-English or non-article/review publications were not considered, which may result in the omission of relevant publications. Secondly, the use of a single database source (WoS) and specific search parameters may have led to the exclusion of certain publications. Future research should consider using various databases and broader definitions of terms to collect data. Lastly, due to the constant updates in databases and the evaluation limited to publications from 2000 to 2022, there is a possibility that the current state of publications may not be fully represented, leading to discrepancies between bibliometric findings and the actual state of publications. Hence, future studies will employ more advanced methods such as machine learning and text mining for comprehensive analysis.

## Data availability statement

The original contributions presented in the study are included in the article/supplementary material, further inquiries can be directed to the corresponding author.

## Author contributions

WT: Software, Visualization, Writing – original draft. YaC: Methodology, Visualization, Writing – original draft. LM: Writing – original draft. YuC: Software, Writing – original draft. BY: Methodology, Writing – review & editing. RL: Data curation, Formal analysis, Writing – review & editing. ZL: Project administration, Software, Writing – review & editing. YW: Methodology, Project administration, Writing – review & editing. XW: Formal analysis, Methodology, Writing – review & editing. XG: Formal analysis, Investigation, Writing – review & editing. WZ: Data curation, Methodology, Writing – review & editing. XC: Data curation, Software, Writing – review & editing. ML: Software, Writing – review & editing. YZ: Formal analysis, Supervision, Writing – review & editing. GG: Conceptualization, Funding acquisition, Writing – review & editing.

## References

[1] Lawton MT Rutledge WC Kim H Stapf C Whitehead KJ Li DY. Brain arteriovenous malformations. Nat Rev Dis Primers. (2015) 1:15008. doi: 10.1038/nrdp.2015.8 27188382

[2] Halim AX Johnston SC Singh V McCulloch CE Bennett JP Achrol AS. Longitudinal risk of intracranial hemorrhage in patients with arteriovenous malformation of the brain within a defined population. Stroke. (2004) 35:1697–702. doi: 10.1161/01.Str.0000130988.44824.29, PMID: 15166396

[3] Stapf C Mast H Sciacca RR Choi JH Khaw AV Connolly ES. Predictors of hemorrhage in patients with untreated brain arteriovenous malformation. Neurology. (2006) 66:1350–5. doi: 10.1212/01.wnl.0000210524.68507.87, PMID: 16682666

[4] Ogilvy CS Stieg PE Awad I Brown RD Jr Kondziolka D Rosenwasser R. Aha scientific statement: recommendations for the management of intracranial arteriovenous malformations: a statement for healthcare professionals from a special writing group of the Stroke Council, American Stroke Association. Stroke. (2001) 32:1458–71. doi: 10.1161/01.str.32.6.1458, PMID: 11387517

[5] Brown RD Jr Wiebers DO Forbes G O’Fallon WM Piepgras DG Marsh WR. The natural history of Unruptured intracranial arteriovenous malformations. J Neurosurg. (1988) 68:352–7. doi: 10.3171/jns.1988.68.3.0352 3343606

[6] Derdeyn CP Zipfel GJ Albuquerque FC Cooke DL Feldmann E Sheehan JP. Management of brain arteriovenous malformations: a scientific statement for healthcare professionals from the American Heart Association/American Stroke Association. Stroke. (2017) 48:e200–24. doi: 10.1161/str.0000000000000134, PMID: 28642352

[7] Agarwal A Durairajanayagam D Tatagari S Esteves SC Harlev A Henkel R. Bibliometrics: tracking research impact by selecting the appropriate metrics. Asian J Androl. (2016) 18:296–309. doi: 10.4103/1008-682x.171582, PMID: 26806079 PMC4770502

[8] Zhang L Ling J Lin M. Artificial intelligence in renewable energy: a comprehensive bibliometric analysis. Energy Rep. (2022) 8:14072–88. doi: 10.1016/j.egyr.2022.10.347

[9] Zhong M Lin M. Bibliometric analysis for economy in Covid-19 pandemic. Heliyon. (2022) 8:e10757. doi: 10.1016/j.heliyon.2022.e10757, PMID: 36185135 PMC9509534

[10] Chen Y Lin M Zhuang D. Wastewater treatment and emerging contaminants: bibliometric analysis. Chemosphere. (2022) 297:133932. doi: 10.1016/j.chemosphere.2022.133932, PMID: 35149018

[11] Zhang L Ling J Lin M. Carbon neutrality: a comprehensive bibliometric analysis. Environ Sci Pollut Res Int. (2023) 30:45498–514. Epub 2023/02/18. doi: 10.1007/s11356-023-25797-w, PMID: 36800084

[12] Yu D He X. A bibliometric study for Dea applied to energy efficiency: trends and future challenges. Appl Energy. (2020) 268:115048. doi: 10.1016/j.apenergy.2020.115048

[13] Burney IA Aal Hamad AH Hashmi SFA Ahmad N Pervez N. Evolution of the Management of Brain Metastases: a bibliometric analysis. Cancers (Basel). (2023) 15:5570. doi: 10.3390/cancers15235570 38067273 PMC10705608

[14] Yang R Ren Y Maingard J Thijs V Le DVA Kok HK. The 100 Most cited articles in the endovascular treatment of brain arteriovenous malformations. Brain Circulat. (2021) 7:49–64. doi: 10.4103/bc.bc_46_20, PMID: 34189347 PMC8191531

[15] Ramos MB Teixeira MJ Preul MC Spetzler RF Figueiredo EG. A bibliometric study of the most cited reports in central nervous system arteriovenous malformations. World Neurosurg. (2019) 129:261–8. doi: 10.1016/j.wneu.2019.06.048, PMID: 31207367

[16] Aria M Cuccurullo C. Bibliometrix: an R-tool for comprehensive science mapping analysis. J Informet. (2017) 11:959–75. doi: 10.1016/j.joi.2017.08.007

[17] van Eck NJ Waltman L. Software survey: Vosviewer, a computer program for bibliometric mapping. Scientometrics. (2010) 84:523–38. doi: 10.1007/s11192-009-0146-3, PMID: 20585380 PMC2883932

[18] Synnestvedt MB Chen C Holmes JH. Citespace ii: visualization and knowledge discovery in bibliographic databases. AMIA Annual Symposium proceedings AMIA Symposium. (2005) 2005:724–8. 16779135 PMC1560567

[19] Mohr JP Parides MK Stapf C Moquete E Moy CS Overbey JR. Medical management with or without interventional therapy for Unruptured brain arteriovenous malformations (Aruba): a multicentre, non-blinded, randomised trial. Lancet. (2014) 383:614–21. doi: 10.1016/s0140-6736(13)62302-8, PMID: 24268105 PMC4119885

[20] Pardali E Ten Dijke P. Tgfβ signaling and cardiovascular diseases. Int J Biol Sci. (2012) 8:195–213. doi: 10.7150/ijbs.3805 22253564 PMC3258560

[21] Leblanc GG Golanov E Awad IA Young WL. Biology of vascular malformations of the brain. Stroke. (2009) 40:e694–702. doi: 10.1161/strokeaha.109.563692, PMID: 19834013 PMC2810509

[22] Crist AM Lee AR Patel NR Westhoff DE Meadows SM. Vascular deficiency of Smad4 causes arteriovenous malformations: a mouse model of hereditary hemorrhagic telangiectasia. Angiogenesis. (2018) 21:363–80. doi: 10.1007/s10456-018-9602-0, PMID: 29460088 PMC5878194

[23] Ola R Künzel SH Zhang F Genet G Chakraborty R Pibouin-Fragner L. Smad4 prevents flow induced arteriovenous malformations by inhibiting casein kinase 2. Circulation. (2018) 138:2379–94. doi: 10.1161/circulationaha.118.033842, PMID: 29976569 PMC6309254

[24] Choi EJ Chen W Jun K Arthur HM Young WL Su H. Novel brain arteriovenous malformation mouse models for type 1 hereditary hemorrhagic telangiectasia. PLoS One. (2014) 9:e88511. doi: 10.1371/journal.pone.0088511, PMID: 24520391 PMC3919779

[25] Chen W Sun Z Han Z Jun K Camus M Wankhede M. De novo cerebrovascular malformation in the adult mouse after endothelial Alk1 deletion and Angiogenic stimulation. Stroke. (2014) 45:900–2. doi: 10.1161/strokeaha.113.003655, PMID: 24457293 PMC4117234

[26] Lee HW Xu Y He L Choi W Gonzalez D Jin SW. Role of venous endothelial cells in developmental and pathologic angiogenesis. Circulation. (2021) 144:1308–22. doi: 10.1161/circulationaha.121.054071, PMID: 34474596 PMC9153651

[27] Mahmoud M Allinson KR Zhai Z Oakenfull R Ghandi P Adams RH. Pathogenesis of arteriovenous malformations in the absence of Endoglin. Circ Res. (2010) 106:1425–33. doi: 10.1161/circresaha.109.211037, PMID: 20224041

[28] Walker EJ Su H Shen F Choi EJ Oh SP Chen G. Arteriovenous malformation in the adult mouse brain resembling the human disease. Ann Neurol. (2011) 69:954–62. doi: 10.1002/ana.22348, PMID: 21437931 PMC3117949

[29] Hao Q Zhu Y Su H Shen F Yang GY Kim H. Vegf induces more severe cerebrovascular dysplasia in endoglin than in Alk1 mice. Transl Stroke Res. (2010) 1:197–201. doi: 10.1007/s12975-010-0020-x, PMID: 20640035 PMC2902730

[30] Park SO Wankhede M Lee YJ Choi EJ Fliess N Choe SW. Real-time imaging of de novo arteriovenous malformation in a mouse model of hereditary hemorrhagic telangiectasia. J Clin Invest. (2009) 119:3487–96. doi: 10.1172/jci39482, PMID: 19805914 PMC2769195

[31] Corti P Young S Chen CY Patrick MJ Rochon ER Pekkan K. Interaction between Alk1 and blood flow in the development of arteriovenous malformations. Development. (2011) 138:1573–82. doi: 10.1242/dev.060467, PMID: 21389051 PMC3062425

[32] Drapé E Anquetil T Larrivée B Dubrac A. Brain arteriovenous malformation in hereditary hemorrhagic telangiectasia: recent advances in cellular and molecular mechanisms. Front Hum Neurosci. (2022) 16:1006115. doi: 10.3389/fnhum.2022.1006115, PMID: 36504622 PMC9729275

[33] Nikolaev SI Fish JE Radovanovic I. Somatic activating KRAS mutations in arteriovenous malformations of the brain. N Engl J Med. (2018) 378:1561–2. doi: 10.1056/NEJMc1802190 29669234

[34] Couto JA Huang AY Konczyk DJ Goss JA Fishman SJ Mulliken JB. Somatic Map2k1 mutations are associated with extracranial arteriovenous malformation. Am J Hum Genet. (2017) 100:546–54. doi: 10.1016/j.ajhg.2017.01.018, PMID: 28190454 PMC5339083

[35] Hong T Yan Y Li J Radovanovic I Ma X Shao YW. High prevalence of KRAS/BRAF somatic mutations in brain and spinal cord arteriovenous malformations. Brain. (2019) 142:23–34. doi: 10.1093/brain/awy307, PMID: 30544177

[36] Lindahl P Johansson BR Levéen P Betsholtz C. Pericyte loss and microaneurysm formation in Pdgf-B-deficient mice. Science (New York, NY). (1997) 277:242–5. doi: 10.1126/science.277.5323.242, PMID: 9211853

[37] Shabani Z Schuerger J Su H. Cellular loci involved in the development of brain arteriovenous malformations. Front Hum Neurosci. (2022) 16:968369. doi: 10.3389/fnhum.2022.968369, PMID: 36211120 PMC9532630

[38] Lawson ND Scheer N Pham VN Kim CH Chitnis AB Campos-Ortega JA. Notch signaling is required for arterial-venous differentiation during embryonic vascular development. Development. (2001) 128:3675–83. doi: 10.1242/dev.128.19.3675, PMID: 11585794

[39] Kim YH Hu H Guevara-Gallardo S Lam MT Fong SY Wang RA. Artery and vein size is balanced by Notch and Ephrin B2/Ephb4 during angiogenesis. Development. (2008) 135:3755–64. doi: 10.1242/dev.022475, PMID: 18952909 PMC2596923

[40] Zhong TP Childs S Leu JP Fishman MC. Gridlock Signalling pathway fashions the first embryonic artery. Nature. (2001) 414:216–20. Epub 2001/11/09. doi: 10.1038/35102599, PMID: 11700560

[41] Masumura T Yamamoto K Shimizu N Obi S Ando J. Shear stress increases expression of the arterial endothelial marker ephrinB2 in murine ES cells via the VEGF-Notch signaling pathways. Arterioscler Thromb Vasc Biol. (2009) 29:2125–31. doi: 10.1161/atvbaha.109.193185, PMID: 19797707

[42] Karthika CL Venugopal V Sreelakshmi BJ Krithika S Thomas JM Abraham M. Oscillatory shear stress modulates Notch-mediated endothelial mesenchymal plasticity in cerebral arteriovenous malformations. Cell Mol Biol Lett. (2023) 28:22. doi: 10.1186/s11658-023-00436-x, PMID: 36934253 PMC10024393

[43] Ahn SM Kim YR Kim HN Shin YI Shin HK Choi BT. Electroacupuncture ameliorates memory impairments by enhancing oligodendrocyte regeneration in a mouse model of prolonged cerebral hypoperfusion. Sci Rep. (2016) 6:28646. Epub 2016/06/29. doi: 10.1038/srep28646, PMID: 27350403 PMC4923909

[44] Zhu W Ma L Zhang R Su H. The roles of Endoglin gene in cerebrovascular diseases. Neuroimmunol Neuroinflammat. (2017) 4:199–210. doi: 10.20517/2347-8659.2017.18, PMID: 29098173 PMC5663457

[45] la Sala A Pontecorvo L Agresta A Rosano G Stabile E. Regulation of collateral blood vessel development by the innate and adaptive immune system. Trends Mol Med. (2012) 18:494–501. doi: 10.1016/j.molmed.2012.06.007, PMID: 22818027

[46] Spiller KL Anfang RR Spiller KJ Ng J Nakazawa KR Daulton JW. The role of macrophage phenotype in vascularization of tissue engineering scaffolds. Biomaterials. (2014) 35:4477–88. doi: 10.1016/j.biomaterials.2014.02.012, PMID: 24589361 PMC4000280

[47] Sandoval-Garcia C Yang P Schubert T Schafer S Hetzel S Ahmed A. Comparison of the diagnostic utility of 4d-Dsa with conventional 2d- and 3d-Dsa in the diagnosis of cerebrovascular abnormalities. AJNR Am J Neuroradiol. (2017) 38:729–34. doi: 10.3174/ajnr.A5137, PMID: 28279986 PMC5389915

[48] Sandoval-Garcia C Royalty K Yang P Niemann D Ahmed A Aagaard-Kienitz B. 4D DSA a new technique for arteriovenous malformation evaluation: a feasibility study. J Neurointerv Surg. (2016) 8:300–4. doi: 10.1136/neurintsurg-2014-011534, PMID: 25583531 PMC4740248

[49] Yuki I Ishibashi T Ikemura A Kambayashi Y Kan I Abe Y. O-019 4d digital subtraction angiography: the advantages and limitations in the evaluation of brain arteriovenous malformation and brain aneurysms. J. NeuroIntervent Surg. (2015) 7:A10 – A, A10.2, A1A10. doi: 10.1136/neurintsurg-2015-011917.19

[50] Chen KK Guo WY Yang HC Lin CJ Wu CF Gehrisch S. Application of time-resolved 3D digital subtraction angiography to plan cerebral arteriovenous malformation radiosurgery. AJNR Am J Neuroradiol. (2017) 38:740–6. doi: 10.3174/ajnr.A5074, PMID: 28126751 PMC7960231

[51] Lescher S Gehrisch S Klein S Berkefeld J. Time-resolved 3D rotational angiography: display of detailed neurovascular anatomy in patients with intracranial vascular malformations. J Neurointerv Surg. (2017) 9:887–94. doi: 10.1136/neurintsurg-2016-012462, PMID: 27492375

[52] Klingebiel R Siebert E Diekmann S Wiener E Masuhr F Wagner M. 4-D imaging in cerebrovascular disorders by using 320-slice CT: feasibility and preliminary clinical experience. Acad Radiol. (2009) 16:123–9. doi: 10.1016/j.acra.2008.11.004, PMID: 19124096

[53] Wang H Ye X Gao X Zhou S Lin Z. The diagnosis of arteriovenous malformations by 4D-CTA: a clinical study. J Neuroradiol. (2014) 41:117–23. doi: 10.1016/j.neurad.2013.04.004, PMID: 23774002

[54] Willems PW Taeshineetanakul P Schenk B Brouwer PA Terbrugge KG Krings T. The use of 4D-CTA in the diagnostic work-up of brain arteriovenous malformations. Neuroradiology. (2012) 54:123–31. doi: 10.1007/s00234-011-0864-0, PMID: 21465177 PMC3261398

[55] In’t Veld M Fronczek R Dos Santos MP MAA VW FJA M PWA W. High sensitivity and specificity of 4D-CTA in the detection of cranial arteriovenous shunts. Eur Radiol. (2019) 29:5961–70. Epub 2019/05/16. doi: 10.1007/s00330-019-06234-4, PMID: 31089848 PMC6795637

[56] Hadizadeh DR Kukuk GM Steck DT Gieseke J Urbach H Tschampa HJ. Noninvasive evaluation of cerebral arteriovenous malformations by 4D-MRA for preoperative planning and postoperative follow-up in 56 patients: comparison with DSA and intraoperative findings. AJNR Am J Neuroradiol. (2012) 33:1095–101. doi: 10.3174/ajnr.A2921, PMID: 22300925 PMC8013249

[57] Taschner CA Gieseke J Le Thuc V Rachdi H Reyns N Gauvrit JY. Intracranial arteriovenous malformation: time-resolved contrast-enhanced Mr angiography with combination of parallel imaging, keyhole acquisition, and K-space sampling techniques at 1.5 T. Radiology. (2008) 246:871–9. doi: 10.1148/radiol.2463070293, PMID: 18195381

[58] Yu S Yan L Yao Y Wang S Yang M Wang B. Noncontrast dynamic MRA in intracranial arteriovenous malformation (AVM), comparison with time of flight (TOF) and digital subtraction angiography (DSA). Magn Reson Imaging. (2012) 30:869–77. doi: 10.1016/j.mri.2012.02.027, PMID: 22521994 PMC4143232

[59] Al-Shahi Salman R White PM Counsell CE du Plessis J van Beijnum J Josephson CB. Outcome after conservative management or intervention for unruptured brain arteriovenous malformations. JAMA. (2014) 311:1661–9. doi: 10.1001/jama.2014.3200 24756516

[60] van Beijnum J van der Worp HB Buis DR Al-Shahi Salman R Kappelle LJ Rinkel GJ. Treatment of brain arteriovenous malformations: a systematic review and meta-analysis. JAMA. (2011) 306:2011–9. doi: 10.1001/jama.2011.1632 22068993

[61] Sattari SA Shahbandi A Yang W Feghali J Xu R Huang J. Microsurgery versus microsurgery with preoperative embolization for brain arteriovenous malformation treatment: a systematic review and meta-analysis. Neurosurgery. (2023) 92:27–41. doi: 10.1227/neu.0000000000002171, PMID: 36519858

[62] de Oliveira JG Massella CR Jr de Holanda CVM Giudicissi-Filho M Borba LAB. Microsurgical management of a high-grade brain arteriovenous malformation in the central lobe after unsuccessful radiosurgery. Neurosurg Focus. (2017) 43:V3. doi: 10.3171/2017.7.FocusVid.17128, PMID: 28669269

[63] Paganelli SL Alejandro SA Chang Mulato JE Vela Rojas EJ Dória-Netto HL Campos Filho JM. Microsurgical treatment for a cerebral arteriovenous malformation of the central sulcus. World Neurosurg. (2022) 159:64. doi: 10.1016/j.wneu.2021.12.084, PMID: 34971830

[64] Friedman WA. Stereotactic radiosurgery of intracranial arteriovenous malformations. Neurosurg Clin N Am. (2013) 24:561–74. doi: 10.1016/j.nec.2013.05.002 24093574

[65] Baharvahdat H Blanc R Fahed R Smajda S Ciccio G Desilles JP. Endovascular treatment for low-grade (Spetzler-Martin I-II) brain arteriovenous malformations. AJNR Am J Neuroradiol. (2019) 40:668–72. doi: 10.3174/ajnr.A5988, PMID: 30792251 PMC7048507

[66] Iosif C de Lucena AF Abreu-Mattos LG Ala VHE El-Ghanam A Saleme S. Curative endovascular treatment for low-grade Spetzler-Martin brain arteriovenous malformations: a single-center prospective study. J Neurointerv Surg. (2019) 11:699–705. doi: 10.1136/neurintsurg-2018-014390, PMID: 30602485

[67] Baharvahdat H Blanc R Fahed R Pooyan A Mowla A Escalard S. Endovascular treatment as the main approach for Spetzler-Martin grade iii brain arteriovenous malformations. J Neurointerv Surg. (2021) 13:241–6. doi: 10.1136/neurintsurg-2020-016450, PMID: 32989031

[68] Kato Y Dong VH Chaddad F Takizawa K Izumo T Fukuda H. Expert consensus on the management of brain arteriovenous malformations. Asian J Neurosurg. (2019) 14:1074–81. doi: 10.4103/ajns.AJNS_234_19, PMID: 31903343 PMC6896626

[69] Chen Y Han H Ma L Li R Li Z Yan D. Multimodality treatment for brain arteriovenous malformation in mainland China: design, rationale, and baseline patient characteristics of a nationwide multicenter prospective registry. Chin Neurosurg J. (2022) 8:33. doi: 10.1186/s41016-022-00296-y, PMID: 36253875 PMC9575306

[70] Darsaut TE Magro E Gentric JC Batista AL Chaalala C Roberge D. Treatment of brain AVMS (TOBAS): study protocol for a pragmatic randomized controlled trial. Trials. (2015) 16:497. doi: 10.1186/s13063-015-1019-0, PMID: 26530856 PMC4632683

[71] Nisson PL Fard SA Walter CM Johnstone CM Mooney MA Tayebi Meybodi A. A novel proposed grading system for cerebellar arteriovenous malformations. J Neurosurg. (2019) 132:1105–15. doi: 10.3171/2018.12.Jns181677, PMID: 30849761 PMC6856412

[72] Silva MA Lai PMR Du R Aziz-Sultan MA Patel NJ. The ruptured arteriovenous malformation grading scale (RAGS): an extension of the hunt and hess scale to predict clinical outcome for patients with ruptured brain arteriovenous malformations. Neurosurgery. (2020) 87:193–9. doi: 10.1093/neuros/nyz404, PMID: 31586199

[73] Neidert MC Lawton MT Mader M Seifert B Valavanis A Regli L. The AVICH score: a novel grading system to predict clinical outcome in arteriovenous malformation-related intracerebral hemorrhage. World Neurosurg. (2016) 92:292–7. Epub 2016/05/07. doi: 10.1016/j.wneu.2016.04.080, PMID: 27150647

[74] Jiao Y Lin F Wu J Li H Wang L Jin Z. A supplementary grading scale combining lesion-to-eloquence distance for predicting surgical outcomes of patients with brain arteriovenous malformations. J Neurosurg. (2018) 128:530–40. doi: 10.3171/2016.10.Jns161415, PMID: 28362235

[75] Oermann EK Rubinsteyn A Ding D Mascitelli J Starke RM Bederson JB. Using a machine learning approach to predict outcomes after radiosurgery for cerebral arteriovenous malformations. Sci Rep. (2016) 6:21161. doi: 10.1038/srep21161, PMID: 26856372 PMC4746661

[76] Meng X Gao D He H Sun S Liu A Jin H. A machine learning model predicts the outcome of SRS for residual arteriovenous malformations after partial embolization: a real-world clinical obstacle. World Neurosurg. (2022) 163:e73–82. doi: 10.1016/j.wneu.2022.03.007, PMID: 35276397

[77] Asadi H Kok HK Looby S Brennan P O’Hare A Thornton J. Outcomes and complications after endovascular treatment of brain arteriovenous malformations: a prognostication attempt using artificial intelligence. World Neurosurg. (2016) 96:562–9.e1. doi: 10.1016/j.wneu.2016.09.086, PMID: 27693769

[78] Zhu W Chen W Zou D Wang L Bao C Zhan L. Thalidomide reduces hemorrhage of brain arteriovenous malformations in a mouse model. Stroke. (2018) 49:1232–40. doi: 10.1161/strokeaha.117.020356, PMID: 29593101 PMC5916043

[79] Walker EJ Su H Shen F Degos V Amend G Jun K. Bevacizumab attenuates Vegf-induced angiogenesis and vascular malformations in the adult mouse brain. Stroke. (2012) 43:1925–30. Epub 2012/05/10. doi: 10.1161/strokeaha.111.647982, PMID: 22569934 PMC3404823

[80] Hashimoto T Matsumoto MM Li JF Lawton MT Young WL. Suppression of MMP-9 by doxycycline in brain arteriovenous malformations. BMC Neurol. (2005) 5:1. doi: 10.1186/1471-2377-5-1, PMID: 15667660 PMC547916

